# Painful Sores

**Published:** 1867-12

**Authors:** 


PAINFUL SORES

are very gratefully and speedily relieved by a mixture of morphine and glycerine. But more striking benefits are observed in its use as food, from its inherent nutritious qualities in all that large class of cases where a person needs

